# Macular and optic disc edema and retinal vascular leakage in familial amyloid polyneuropathy with a transthyretin Val30Met mutation: a case report

**DOI:** 10.1186/1752-1947-8-327

**Published:** 2014-10-04

**Authors:** Arnaldo Dias-Santos, Joana Ferreira, João Paulo Cunha

**Affiliations:** 1 Department of Ophthalmology of Central Lisbon Hospital Center, Lisbon, Portugal; 2 Investigation Ophthalmology Lisbon Center, Lisbon, Portugal; 3 Hospital de Santo António dos Capuchos, Serviço de Oftalmologia, Alameda de Santo António dos Capuchos, 1169-050 Lisbon, Portugal

**Keywords:** Cystoid macular edema, Familial amyloid polyneuropathy, Intravitreal dexamethasone implant, Laser panretinal photocoagulation, Optic disc edema, Retinal vascular leakage

## Abstract

**Introduction:**

Familial amyloid polyneuropathy is a group of autosomal dominant disorders characterized by extracellular amyloid deposition in several target organs. This paper aims to report an unusual manifestation of retinal vascular leakage including optic disc and macular edema in a patient with familial amyloid polyneuropathy.

**Case presentation:**

A 37-year-old Portuguese Caucasian man with Val30Met transthyretin-related familial amyloid polyneuropathy presented with rapidly progressing visual loss in his left eye. He had undergone liver transplantation at the age of 30 with neurologic stabilization. Fundoscopy and fluorescein angiogram revealed optic disc and macular edema as well as vessel wall staining with leakage in the posterior pole and mid-periphery, without vitreous opacities. A diagnostic work-up for infectious, autoimmune and neoplasic conditions was negative. Systemic immunosuppression was increased but without improvement. Sustained resolution of macular edema was observed after intravitreal injection of dexamethasone implant and laser panretinal photocoagulation.

**Conclusions:**

To the best of our knowledge, this is the first report of a rare ocular manifestation of familial amyloid polyneuropathy which represents a new therapeutic challenge. Intravitreal injection of sustained release dexamethasone implant and panretinal photocoagulation may be an effective eye-saving therapeutic approach.

## Introduction

Familial amyloid polyneuropathy (FAP) or paramyloidosis is a heterogenous group of autosomal dominant disorders characterized by extracellular amyloid deposition in peripheral nerves, cardiac muscle, kidneys and eyes [1]. There are three precursor proteins that can give rise to amyloid production: transthyretin (TTR), apolipoprotein A-1 and gelsolin, each resulting in a distinct type of FAP [2]. TTR-related FAP is the most frequent type and more than 80 mutations involving this protein have been described [3,4]. A single amino acid substitution of valine for methionine at position 30 of TTR (Val30Met) is the most frequent TTR-related FAP causing mutation [5]. The liver is the major site of synthesis of circulating TTR [6], but there is also evidence of TTR production in other tissues like the retinal pigment epithelium, the choroid plexus of the brain and the visceral yolk sac endoderm [7]. Since 1990, liver transplantation has been considered the main therapeutic approach, halting the progression of neurologic complications [8]. However, ocular complications have been reported to continue or even increase after liver transplantation [9,10]. The most frequently reported ocular manifestations of FAP are keratoconjunctivitis sicca, abnormal conjunctival vessels, pupillary abnormalities, glaucoma and vitreous opacities [11].

The purpose of this paper is to report a case of retinal vascular leakage including optic disc and cystoid macular edema in a patient with Val30Met TTR-related FAP.

## Case presentation

**Figure 1:**
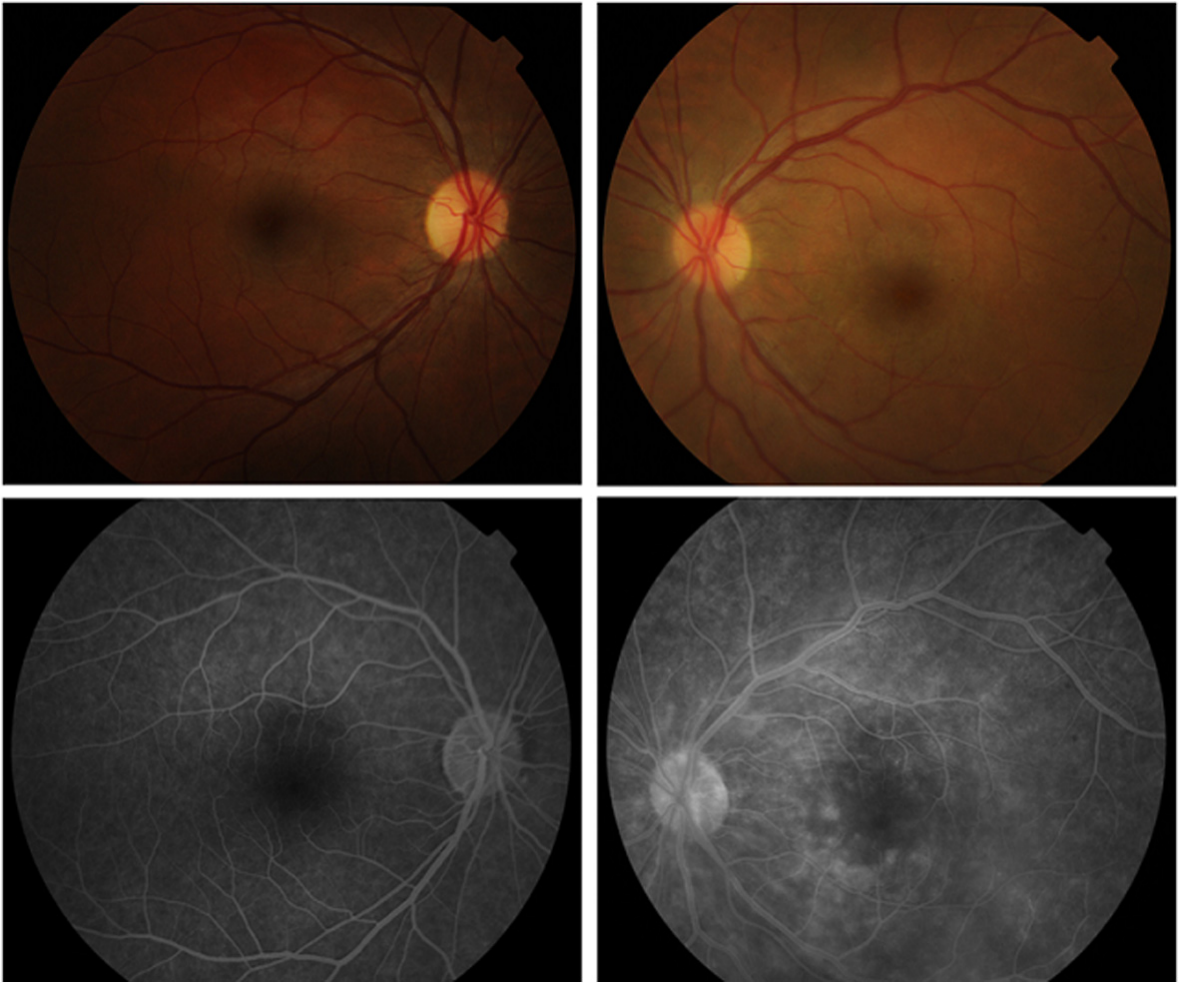
**Baseline retinography and fundus fluorescein angiography**. Right eye retinography (top left) reveals no pathologic alterations. Left eye retinography (top right) showing two dot-blot hemorrhages near the superotemporal vascular arcade, slight optic disc edema as well as macular edema. Right eye fluorescein angiography at 2'38" reveals no changes (bottom left). Left eye fluorescein angiography at 3'37" reveals optic disc and macular edema as well as vascular fluorescein leakage and vessel wall staining (bottom right).

**Figure 2:**
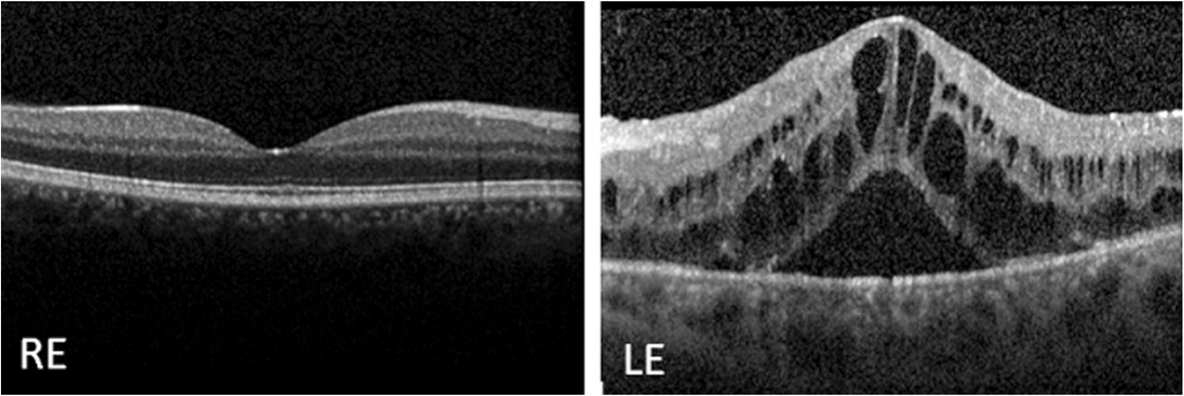
**Baseline macular spectral domain optical coherence tomography.** The examination reveals a normal macular morphology in the right eye and cystoid macular edema in the left eye. Abbreviations: LE, left eye; RE, right eye.

A 37-year-old Portuguese Caucasian man diagnosed with Val30Met FAP 12 years previously presented with painless, rapidly progressive visual loss in his left eye (LE). He also had sensory-motor polyneuropathy, gastrointestinal dysmotility and bradyarrhythmia which stabilized after pacemaker implantation and liver transplantation at the age of 30. He was medicated with prednisolone (5mg/day), tacrolimus (3mg/day), sirolimus (2mg/day), mycophenolate mofetil (1500mg/day), ursodeoxycholic acid and pantoprazole. There was no other relevant past medical history. His visual acuity was 20/20 in his right eye (RE) and 20/200 in his LE. Slit lamp biomicroscopy was normal and intraocular pressure (IOP) was 15mmHg in both eyes. A fundus examination after pupillary dilatation was normal in his RE, whereas two dot-blot hemorrhages near his superotemporal vascular arcade, slight optic disc edema as well as macular edema were identified in his LE (Figure 1). No vitreous opacities were observed and his retinal periphery was normal bilaterally. Fluorescein angiography (FA) confirmed LE optic disc edema and showed vascular fluorescein leakage and vessel wall staining in the posterior pole and mid-periphery, without capillary exclusion zones or evidence of neovascularization. LE macular edema was also evident on FA. Macular optical coherence tomography (OCT) revealed a normal morphology in his RE and cystoid macular edema in his LE (Figure 2).

**Figure 3:**
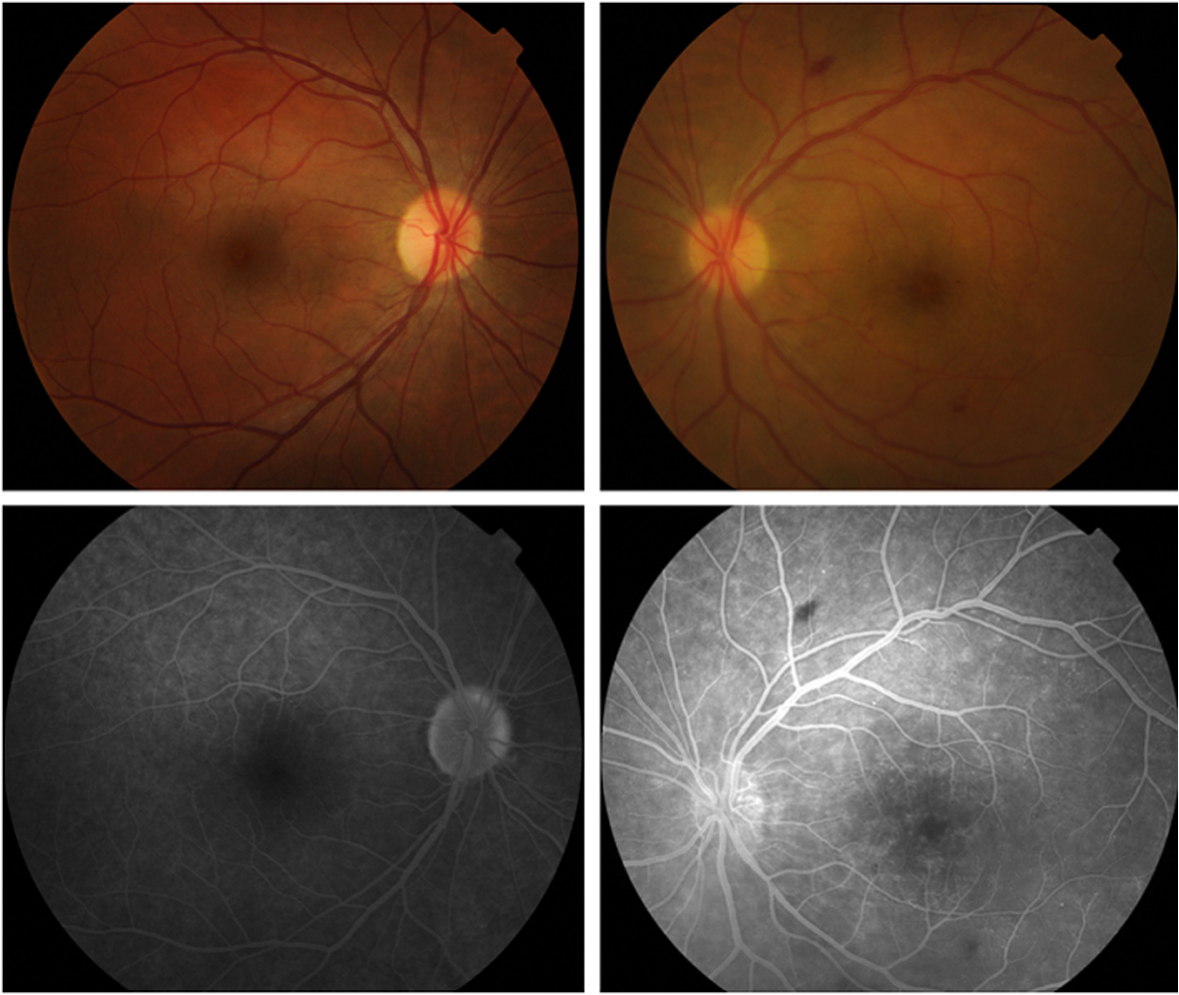
**Retinography and fundus fluorescein angiography following therapy with intravitreal bevacizumab.** Right eye retinography (top left) reveals no pathologic alterations. Left eye retinography (top right) reveals optic disc and macular edema as well as a dot-blot hemorrhage near the inferotemporal arcade and a mid-peripheral flame-shaped hemorrhage. Right eye fluorescein angiography at 4'00" reveals staining of the optic disc margins (bottom left). Left eye fluorescein angiography at 3'30" reveals a reduction in macular and perivascular exudation in comparison to the previous examination; optic disc edema is also evident (bottom right).

**Figure 4:**
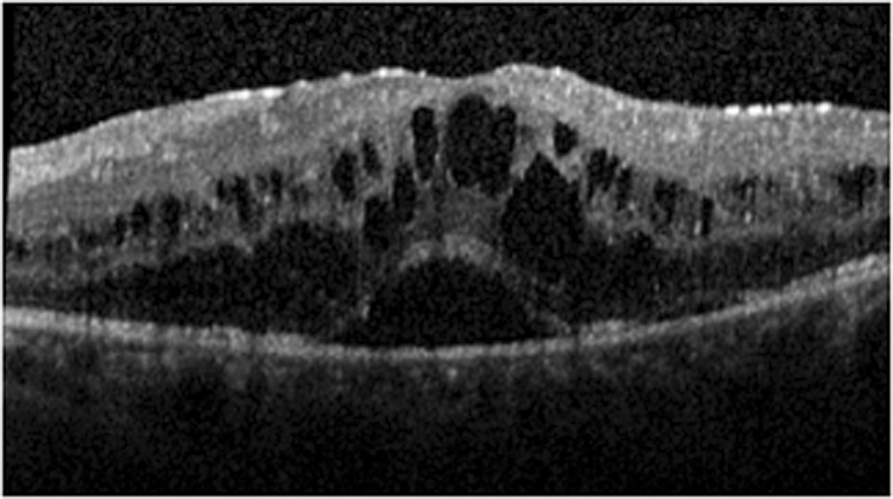
Macular spectral domain optical coherence tomography of the left eye following therapy with intravitreal bevacizumab.

**Figure 5:**
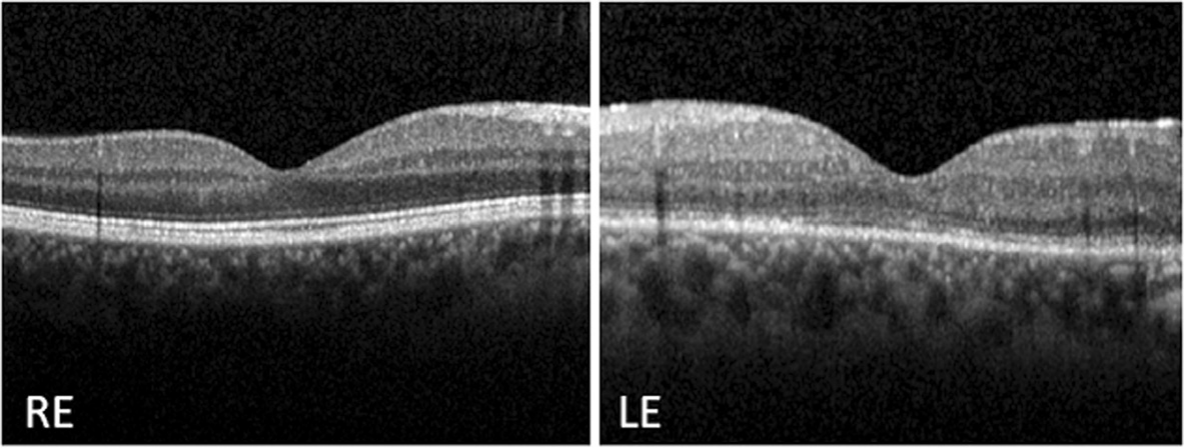
**Macular spectral domain optical coherence tomography following intravitreal dexamethasone implant 0.7mg and laser panretinal photocoagulation in the left eye.** The examination reveals a complete resolution of the macular edema in the left eye. The right eye presented normal macular morphology. Abbreviations: LE, left eye; RE, right eye.

A primary extensive work-up for systemic infectious, autoimmune, neoplasic and inflammatory conditions was performed including complete blood profile, chest X-ray, serologic tests (toxoplasmosis, syphilis, human immunodeficiency virus, hepatitis, cytomegalovirus) and other investigations for tuberculosis (purified protein derivative skin test and sputum analysis), fungi, acid-alcohol resistant bacilli and bacteria. No clinically remarkable findings were observed. Liver function tests were also unremarkable (aspartate aminotransferase 27U/L, alanine aminotransferase 30U/L, gamma-glutamyl transferase 26U/L, alkaline phosphatase 132U/L, lactate dehydrogenase 421U/L, total bilirubin 1.30mg/dL, albumin 4.40g/dL). Brain and orbital magnetic resonance imaging results were also normal.Prednisolone dosage was increased to 60mg/day but no clinical improvement was observed. This was followed by two intravitreal injections of bevacizumab, which resulted in slight but fugacious clinical and functional improvement. At this time his visual acuity was 20/20 in his RE and 20/200 in his LE. FA and OCT revealed only a slight reduction in macular edema and perivascular exudation in his LE (Figures 3 and 4). The authors then decided to perform an intravitreal injection of triamcinolone in his LE and obtained a substantial improvement in macular edema which, however, recurred 6 weeks after the injection. Given the positive response to intravitreal triamcinolone, dexamethasone intravitreal implant 0.7mg (Ozurdex®; Allergan, Irvine, CA, USA) was performed, followed 2 weeks later by laser panretinal photocoagulation (LPP) in his LE. One month after completing LPP, his visual acuity was 20/20 in his RE and 20/50 in his LE. The IOP was 16mmHg in his RE and 30mmHg in his LE; the control of IOP was further achieved with a fixed combination of 2% dorzolamide/0.5% timolol. Macular OCT revealed a complete resolution of macular edema at this stage (Figure 5).

## Conclusions

Here we describe unusual ocular findings in a patient with paramyloidosis. FAP-related retinal vascular leakage is rare and it is usually associated with amyloid vitreous deposits, which were not observed in this patient. Causes of ocular inflammation were excluded with an extensive diagnostic work-up. We cannot exclude the hypothesis of toxic microangiopathy related to drugs such as sirolimus or tacrolimus. However, the limitation of this reaction to the eye and the positive response to local intravitreal therapy favors, in our opinion, the hypothesis of FAP-related ocular complications. Thus, this is probably the first report of optic disc edema, retinal vascular leakage and cystoid macular edema in a patient with FAP.

Amyloid deposition within the walls of retinal vessels had been previously described. It has been hypothesized that this impregnation may lead to retinal ischemia and vessel wall weakening which would ultimately lead to perivascular exudation as well as retinal and vitreous hemorrhages [12]. O’Hearn *et al*. reported that vitreous vascular endothelial growth factor (VEGF) levels in FAP patients were raised to similar levels as those found in patients with inactive proliferative diabetic retinopathy [13]. Retinal neovascularization is a rare complication of this disease, supporting the important role of VEGF upregulation in its pathogenesis [11]. Zou and colleagues also reported elevated levels of VEGF in vitreous samples and in the serum of patients with ocular complications of TTR-related FAP [14]. Such elevated vitreous VEGF levels could also account for the macular edema observed in our patient. In this patient we observed a positive response to both anti-VEGF and triamcinolone intravitreal injection; however, the effect was more pronounced following triamcinolone. A more sustained response was obtained following intravitreal injection of dexamethasone implant. To date there are no other reports or studies regarding the efficacy of intravitreal anti-VEGF or corticosteroids for the treatment of ocular complications of FAP.

LPP therapy has been recently proposed to treat ocular complications of FAP. During 3 years of follow up, LPP prevented the progression of vitreous and retinal amyloid deposits in two patients [15]. After liver transplantation, the retinal pigment epithelium plays the major role in ocular amyloid production. Therefore, the destruction of this tissue by retinal photocoagulation would theoretically decrease the production of abnormal TTR in the eye. In our patient we performed LPP in his LE in an attempt to halt the natural history of the disease and, at the same time, to try to reduce the burden of intravitreal injections.

As a result of a longer life expectancy in patients with FAP obtained with liver transplantation, more frequent and more complex ocular complications are expected to appear in our clinical practice. Intravitreal corticosteroid therapy in isolation or combined with laser photocoagulation is a promising therapeutic approach to these patients. However, additional and larger multicenter studies are needed to evaluate the efficacy and safety of these therapies in the management of ocular manifestations of FAP.

## Consent

Written informed consent was obtained from the patient for publication of this case report and accompanying images. A copy of the written consent is available for review by the Editor-in-Chief of this journal.

## Competing interests

The authors declare that they have no competing interests.

## Authors’ contributions

ADS analyzed and interpreted the patient’s data regarding the disease and was a major contributor in writing the manuscript. JF and JPC analyzed and interpreted the patient’s data. All authors read and approved the final manuscript.
